# Effect of tobacco control policies on intention to quit smoking cigarettes: A study from Beirut, Lebanon

**DOI:** 10.18332/tid/111128

**Published:** 2019-09-02

**Authors:** Monique Chaaya, Rima Nakkash, Dahlia Saab, Lina Kadi, Rima Afifi

**Affiliations:** 1 Department of Epidemiology and Population Health, American University of Beirut, Beirut, Lebanon; 2 Department of Health Promotion and Community Health, American University of Beirut, Beirut, Lebanon; 3 Department of Community and Behavioral Health, The University of Iowa, Iowa City, United States

**Keywords:** tobacco control, tobacco policy, intention to quit smoking, Lebanon

## Abstract

**INTRODUCTION:**

In Lebanon, the tobacco control policy, Law 174, became effective in 2011. Using the International Tobacco Control Policy Evaluation Project (ITC) conceptual model, this study aims to assess the association between exposure to control measures related to the policy and the intention to quit, which is a mediator in the pathway leading to behavioral change (quitting).

**METHODS:**

This is a secondary data analysis of 154 cigarette smokers from a cross-sectional survey that assessed compliance with Law 174 among Beirut residents aged 15–65 years. Data were collected face-to-face, three months after the implementation of indoor public places and tobacco advertisement/promotion bans. Intention to quit smoking was the main outcome. Exposure to policy control measures such as seeing smokers in restaurants, and noticing warning labels on cigarette packs were the explanatory variables. Sociodemographics, past smoking behavior, and psychosocial variables were also considered for their moderating and mediating effects, respectively. Crude and adjusted odds ratios (ORs) were generated. Sobel test was used to check for possible mediation.

**RESULTS:**

Intention to quit was reported by 24% of cigarette smokers. The association between noticing warning labels and having intentions to quit was statistically significant (adjusted OR=6.27). Concerns about influencing children’s smoking behavior had a statistically significant mediation effect on the relationship. After adding the interaction term between noticing the warnings and previous quit attempts, the OR was inflated to 12.92, suggesting a possible interaction.

**CONCLUSIONS:**

This study offers preliminary insight into how Lebanese smokers are influenced by policy related control measures like health warning labels on cigarette packs. Tobacco control policy advocates should push for stronger enforcement of public smoking bans in general. Behavioral intervention should work on the mediator variables to influence smoking behavior and encourage quitting. Further prospective studies modelling quitting as outcome are needed.

## INTRODUCTION

According to the World Health Organization (WHO), one in ten deaths worldwide are attributed to tobacco use^1,2^. Tobacco also affects those exposed to secondhand smoke yielding a yearly death toll of almost 600000 people^1^. The World Health Organization Framework Convention on Tobacco Control (WHO-FCTC) proposes a set of evidence-based tobacco control policies that ratifying countries are obligated to adopt in order to prevent and control this epidemic. Previous research has shown that the most effective tobacco control interventions are those that target policies at the population level^2^. These include implementing indoor smoking bans, increasing taxes on tobacco products, banning tobacco advertisements, mandating larger textual and pictorial health warnings, and implementing strong population-based cessation strategies^2^. Such policies are expected to protect non-smokers from the harms of tobacco use and to contribute to smokers’ behavior change, including quitting.

Smoking cessation is associated with numerous health benefits for smokers and non-smokers^3^. Although 40–75% of current smokers from various countries intend to quit and 30–55% have attempted quitting in the past year^2^, a very small proportion achieve long-term abstinence^4^. There is a growing body of evidence suggesting that tobacco control policies have been effective in enhancing smoking cessation rates. For example, the presence of health warning (HW) labels on cigarette packs influences an individual’s intention to quit and quitting behavior^5^. Indoor smoking bans and policies controlling media messages have also been reported to have an impact on quitting^6,7^. The Global Adult Tobacco Survey (GATS) data, collected from 17 countries, clearly indicate an association between intention to quit smoking and anti-smoking messages in media channels^8^. These findings were also echoed by a study conducted in India^9^.

Shortly after the FCTC ratification, the International Tobacco Control Policy Evaluation Project (ITC Project) was developed as the first international cohort study that measures the impact of policies on behaviors. Based on a conceptual model, the ITC uses prospective surveys to assess the influence of tobacco control policies on psychosocial mediators and behavior. Results are used to promote strong evidence-based policies at the country level^10^. The ITC conceptual model explains how policy-related variables (such as label salience and perceived cost) influence smoking behavior (quitting) through psychosocial mediators (perceived risk and severity) taking into account moderators and confounders (such as country, sociodemographics and personality)^10^.

In Lebanon, the smoking prevalence is high compared to other middle-income countries reaching 34% in males and 21.2% in females for daily cigarette consumption and 26.5% and 24.3%, respectively, for waterpipe consumption^11^. The estimated annual loss attributed to smoking is US$326.7 million (equivalent to 1.1% of the GDP) including costs of treatment from smoking-related illnesses, loss of work productivity, as well as environmental degradation costs^12^.

Although Lebanon ratified the FCTC in 2005, its first tobacco control legislation was not adopted until 2011^13^. Law 174 banned tobacco advertising and smoking in all indoor public places, prohibited the promotion and distribution of free samples of tobacco products, as well as the use of misleading elements such as ‘light’, and ‘ultra-light’ and required larger text and pictorial health warning labels on tobacco product packaging^14^. The advertising and sponsorship took effect immediately (August 2011), however, the indoor smoking ban in the hospitality sector became effective one year later (September 2012). Similarly, a decree to mandate larger textual warnings was also issued in September 2012 but was enforced the following year. A study, assessing compliance three months after the implementation of the policy , found that overall there was good compliance with the ban by the advertising sector, however, the compliance with the indoor smoking ban was much more variable^14^. Policy and decision makers support for Law 174 has waned in the face of strong industry and hospitality sector opposition. Therefore, local research providing evidence for the impact of smoking on the health and economic sectors in Lebanon is needed to support continued advocacy for the law. In the absence of prospective data and long-term surveillance of the population health status, evidence generated from cross-sectional studies, on the association between exposure to policy and smoking behavior (such as quitting) could serve as proxy for the effect of tobacco policies.

The aim of the current study is to assess the association between exposure to specific control measures related to Law 174 and intentions to quit cigarette smoking, using the ITC Project conceptual model as a guide^10^. Intention not to smoke is a first step towards quitting, the ultimate outcome for change in smoking behavior.

## METHODS

### Study design and sample

This study is a secondary analysis of data collected between December 2012 and March 2013, through a population-based cross-sectional survey that aimed at assessing compliance with tobacco control policies among smokers and non-smokers residing in Beirut (the capital of Lebanon). The study was conducted almost one year after the smoking ban in restaurants, nightclubs, hotels and other tourism venues went into effect. Noteworthy, the hospitality sector was given a grace period of one year following the adoption of the law (2011), however, the indoor smoking ban in all other public places and the tobacco advertisement and promotion ban went into effect immediately.

A random sample of 159 households was selected out of which a sample of 468 respondents, aged 15–65 years, participated in the study (154 cigarette smokers, 138 waterpipe smokers and 176 nonsmokers). The households were identified following a multistage cluster sampling whereby a random sample of the city zones was chosen, and in each zone a random number of blocks was selected. Buildings in selected blocks were mapped and systematic sampling of buildings and households was performed. In each sampled household, a screening roaster was filled to identify the number of household members, their ages and their smoking behavior (cigarette/narghile). Participants older than 18 years were directly considered to take part in the survey; however, a permission was first obtained from parents of younger participants. Surveys were conducted face-to-face using a structured questionnaire, and one type of adult smoker was selected from every household.

Prior to data collection, research members were trained on interviewing techniques including probing and questioning. They also had to complete the Collaborative Institutional Training Initiative (CITI) certification for the ethical conduct of research. The study was approved by the Institutional Review Board of the American University of Beirut. A full description of the data collection methods has been published elsewhere^14^. The ITC Project conceptual framework was used to develop the survey questions, thus included policy-related variables, psychosocial mediators and moderators to study the effect of tobacco control policies on smoking behavior^10^. For the current analysis, only the 154 cigarette smokers were included.

### Measures

The main outcome variable, intention to quit smoking, was measured by the question ‘are you currently planning to quit cigarette smoking?’. Additional data on the time frame and reasons for quitting were collected.

The explanatory variables represented the exposure to two policy-related control measures. The first was noticing health warning messages on cigarette packages by asking: ‘In the last month, how often, if at all, have you noticed health warnings on cigarette packages?’. The second was noticing smoking restrictions in public places by asking if the respondents smoked or saw other people smoking in restaurants, coffee shops, and indoor areas at work (‘Within the past month, the last time you visited a restaurant/coffee shop, were people smoking cigarettes indoor?’).

Psychosocial variables, considered as mediators, included: concerns about influencing children to smoke, beliefs that cigarette smoking has damaged respondents’ health, worries about the future health effects of smoking and concerns about society disapproval. All variables were treated as binary (yes/ no). Questions, such as how often participants saw/ noticed a health warning, originally categorized on a 4-point scale (never to always) were recoded into binary measures (yes/no), due to low response rates in some of the categories.

Other covariates included sociodemographic variables (age, gender, education, marital status, income and working status), and past quit attempts (‘Have you ever attempted to quit cigarette smoking?’).

### Statistical analysis

Frequency distributions were constructed for all variables to check variability, decide on exclusions and determine bracketing. Unadjusted odd ratios (ORs) with 95% confidence intervals (CIs) were used to evaluate the association between the outcome (intention to quit smoking) and all other variables (particularly the ones that were policy-related). Pearson chi-squared test was also used to check for an association between categorical variables.

Multiple logistic regression was conducted to adjust for further possible confounders (such as age and previous quit attempts) in the association between the dependent and the explanatory variables. Only variables with p<0.2 at the bivariate level were included in the multivariable analysis. Testing for interaction between policy-related control measures and the sociodemographic and quit attempt variables was performed by adding an interaction term in the multivariable analysis.

Sobel test was used to check the significance of a possible mediation effect of psychosocial variables in the relationship between policy-related variables and the outcome of interest. All analyses were performed on weighted data to adjust for sampling imbalance between the clusters. All statistical tests with p<0.05 were considered statistically significant. Analysis was performed using Stata 10 for Windows.

## RESULTS

### Sample characteristics

The sample, consisting of 154 cigarette smokers, was characterized by the predominance of males (55.9%) and participants aged ≥40 years (61.5%). At the time of the study, the majority of respondents were ever married (76.7%), had at least a secondary or a technical education (68%), were working (58.7%) and were earning more than LBP 1.5 million/month, equivalent to US$1000 (65.6%) (Table 1).

**Table 1:** Sociodemographic characteristics of the sample and unadjusted odds ratios (OR) of the association between intention to quit smoking and sociodemographic characteristics (N=154 )

*Variables*	*N*	*%*	*OR*	*95% CI*	p*
**Age** (years)					
15–29	41	22.8	1		
30–39	23	15.8	6.45	(1.63–25.62)	0.008
≥40	90	61.5	2.89	(0.89–9.41)	0.977
**Gender**					
Male	84	55.9	1		
Female	70	44.1	1.76	(0.61–5.14)	0.296
**Educational status**					
Primary/Intermediate	58	31.9	1		
Secondary/Technical	50	30.7	2.09	(0.78–5.65)	0.144
University	45	37.4	1.53	(0.42–5.61)	0.521
**Marital status**					
Ever married	112	76.7	1		
Never married	35	23.3	0.77	(0.21–2.89)	0.702
**Monthly household income** (million LBP)					
0.5 to <1.5	57	34.4	1		
≥1.5	89	65.6	1.19	(0.46–3.06)	0.718
**Currently working**					
Yes	82	58.7	1		
No	68	41.3	1.19	(0.42–3.32)	0.742

* Based on chi-squared tests. LBP: Lebanese Pounds.

About one fourth of the surveyed cigarette smokers reported intentions to quit at the time of the survey (24%), yet less than 10% had set a time frame for quitting. The common reasons behind the intentions to quit were: having health problems (97%), worrying about the health effects of smoking (74%) and setting a good example to children (55%).

### Exposure to policies

With regard to exposure to the policy related control measures, 74.9% of participants reported noticing the health warning messages on the cigarette packages and more than 50% reported being exposed to smoking in public places, mainly in restaurants and at work (Table 2).

**Table 2:** Unadjusted odds ratios (OR) of the association between intention to quit smoking and policy specific variables (N=154 )

*Policy-specific variables*	*N*	*%*	*OR*	*95% CI*	p*
Notice warning labels					
Yes	109	74.9	7.60	(2.02–28.60)	0.003
No	45	25.1	1		
Notice smoking restriction in restaurants/coffee shops (not seeing people smoking indoors)					
Yes	63	57.7	1.95	(0.65–5.85)	0.233
No	63	42.3	1		
Notice smoking restriction in restaurants/coffee shops (participant not smoking indoors)					
Yes	72	56.7	0.56	(0.18–1.75)	0.314
No	55	43.3	1		
Notice smoking restriction at workplaces					
Yes	42	56.1	1.37	(0.33–5.66)	0.658
No	45	43.9	1		

* Based on chi-squared tests.

### Factors associated with intention to quit smoking: bivariate associations

Table 2 examines the bivariate association between intention to quit smoking and selected policy-related variables. Noticing warning labels on cigarette packs was the only variable statistically significantly associated with intention to quit smoking (OR=7.6, 95% CI: 2.02–28.60). As for the sociodemographic variables, only age was statistically significantly associated with the latter, especially those aged 30–39 years (OR=6.45, 95% CI: 1.63–25.62) compared to people aged 15–29 years (Table 1).

With regard to psychosocial variables, only respondents who had concerns about influencing their children were likely to report intention to quit smoking (OR=7.38, 95% CI: 2.41–22.65). Other psychosocial attitude questions were not statistically significantly associated with the outcome. Finally, the odds of intending to quit among participants who had previous quit attempts were 4 times (95% CI: 1.55– 12.37) the odds of those who had never attempted (Table 3).

**Table 3:** Unadjusted odds ratios (OR) of the association between intention to quit smoking and psychosocial characteristics and ever quit attempt (N=154 )

*Variables*	*N*	*%*	*OR*	*95% CI*	p*
**Psychosocial variables**					
Worry to influence children to smoke					
Yes	92	65.2	7.38	(2.41–22.65)	0.001
No	62	34.8	1		
Damaged health					
Yes	100	67.2	0.98	(0.34–2.84)	0.971
No	51	32.8	1		
Worry to damage health					
Yes	111	75.0	0.84	(0.27–2.68)	0.771
No	38	25.0	1		
Society disapproves smoking					
Yes	88	51.2	0.650	(0.24–1.75)	0.390
No	66	48.8	1		
**Past behavior variables**					
Ever attempted quitting smoking					
Yes	45	34.0	4.38	(1.55–12.37)	0.006
No	108	66.0	1		

* Based on chi-squared tests.

### Mediating effects

‘Worry about influencing children to smoke cigarettes’ had a 37.2% mediation effect on the relationship between noticing the warning label on cigarette packs and the intention to quit smoking (p=0.010).

### Multivariate results

Table 4 shows the adjusted OR (AOR) for the relationship between intention to quit smoking and noticing the warning label, considering age as a confounder (Model 1). Model 2 includes the same variables in addition to the mediator. After controlling for age, the magnitude of the association of intention to quit smoking with noticing the warning label on cigarette packs did not change and remained statistically significant. However, after adding the mediator variable ‘worry about influencing children’ to the earlier model, the independent variable remained statistically significant but the AOR decreased from 7.86 to 4.88, clearly indicating a mediation effect.

**Table 4:** Multivariable logistic regression of intention to quit smoking with the main policy-specific variable ‘noticing the warning label’, controlling for the mediator variable ‘worrying about the influence on children to smoke’ and age (N=154 )

	*Model 1 (without mediator)*			*Model 2 (with mediator)*		
	*AOR*	*95% CI*	*p*	*AOR*	*95% CI*	*p*
**Exposure**						
Notice warning labels						
Yes	7.86	(2.20–28.09)	0.002	4.88	(1.29–18.53)	0.02
No	1			1		
**Covariates**						
Worry about the influence on children to smoke						
Yes				3.59	(1.05–12.23)	0.042
No				1		
**Age** (years)						
15–29	1			1		
30–39	6.85	(1.61–29.13)	0.009	4.18	(0.94–18.63)	0.061
≥40	2.84	(0.85–9.49)	0.089	2.22	(0.66–7.52)	0.198

AOR: adjusted odds ratio.

Model 3 includes the main independent variable controlling for age and previous quit attempts (moderator). Model 4 is the same as Model 3 but includes the interaction term between previous attempts to quit and noticing the warning labels (Table 5). After adjusting for age and previous quit attempts, the association between intention to quit smoking and noticing the warning labels remained statistically significant (Model 3). However, after adding the interaction term to the same model (Model 4), the AOR was inflated to 12.92 suggesting a possible interaction between noticing the warning and previously attempting to quit cigarette smoking.

**Table 5:** Multivariable logistic regression of intentions to quit smoking with the main policy-specific variable ‘noticing the warning label’, controlling for the moderator variable ‘previous attempts at quitting smoking’ and age (N=154 )

	*Model 3 (without interaction term)*			*Model 4 (with interaction term)*		
	*AOR*	*95% CI*	*p*	*AOR*	*95% CI*	*p*
**Exposure**						
Notice warning label						
Yes	6.27	(1.79–21.91)	0.004	12.92	(1.50–111.32)	0.02
No	1			1		
**Other covariates**						
Ever attempted quitting smoking						
Yes	2.97	(1.00–8.80)	0.05	12.76	(0.95–172.02)	0.055
No	1			1		
**(Notice warning label) × (Ever attempted quitting)**				0.21	(0.01–3.53)	0.272
**Age** (years)						
15–29	1			1		
30–39	5.17	(1.26–21.22)	0.023	4.69	(1.13–19.49)	0.045
≥40	2.5	(0.75–8.30)	0.133	2.48	(0.74–8.31)	0.14

AOR: adjusted odds ratio.

## DISCUSSION

Guided by the ITC model, we examined how exposure to policy-related variables had an effect on intention to quit cigarette smoking. Despite the very small health warning labels placed on cigarette packs at the time of the evaluation, only ‘noticing warning labels on cigarette packs’ was shown to be statistically significantly associated with intention to quit smoking among surveyed smokers. This is consistent with the literature on the effectiveness of health warning labels and their association with intentions to quit^15–19^. Although this finding cannot be attributed to the law, as the larger textual warnings had not been implemented at the time of this survey, it should provide more motivation to implement the larger text warnings and to move towards pictorial warnings according to Law 174. Knowing that previous studies on school and college students have suggested a statistically significant effect of textual and pictorial warnings on quit intentions^20^, further research is needed locally to document this impact

The lack of association with other policy related variables is likely a result of a weak implementation of the indoor smoking ban^14^. Literature reports that indoor smoking bans are statistically significantly associated with intention to quit smoking^21,22^. However, in this case, we were measuring a nonintervention, hence the lack of impact was expected and not surprising.

At the bivariate level (unrelated to policies), our findings show that age was statistically significantly associated with intention to quit smoking as older people were more likely to report it. Findings related to this association are mixed in the literature: some studies show that young people have greater intentions to quit^19-22^, while others report that older smokers have higher intentions^23-25^. Other sociodemographic variables such as marital status, education, income, working status and gender were not statistically significantly associated with intention to quit. Although some studies have found links between sociodemographics and quit intentions^9,19,20,26^, a meta-analysis found in the pooled analysis that gender, educational level, income and social class were all not associated with making a quit attempt^25^, which is similar to our findings.

Psychosocial mediators including societal disapproval of smoking, and worrying about damaging one’s own health were not statistically significant predictors of intention to quit. In a systematic review conducted by Vangeli et al.^25^, the only consistent motivational factor for a successful quit attempt was a lower level of cigarette dependence, which we did not measure in our study. The only psychosocial mediator that was associated with intentions to quit was ‘worry about influencing children to smoke’. This is in line with a study by Panda et al.^27^ showing that motivational factors, such as ‘setting a good example for children’ were associated with intentions to quit cigarette smoking. In addition, as supported by a growing body of literature, past quit behavior was found to be a strong determinant of quit intentions^28^.

### Limitations

This study has some limitations. First, data were collected only in Beirut, thus findings may not be generalizable at the national level. Second, the sample included more males, older age groups, and slightly more educated people (compared to the general population). Third, the cross-sectional nature of the study does not allow for any causal associations. Finally, the precision of the estimates was affected when cigarette smokers only (excluding waterpipe smokers) were considered for analysis and this was obvious with very large confidence intervals at the multivariable level.

## CONCLUSIONS

Our study is the first in Lebanon to examine the implementation and effectiveness of control measures related to tobacco policy. It is critical to note that a lack of association between these measures and intentions to quit smoking is mainly due to a lack of policy enforcement rather than a lack of impact. The findings suggest that health warning labels influence smoking behavior, thus, policy makers should be given an impetus to place larger text and pictorial health warnings on cigarette packs. Similar cross-sectional studies are essential to document compliance with the law. Longitudinal studies are also needed to assess the implementation process, give feedbacks to policy makers, and advocate for stronger enforcement. Only then, we may start to see the positive impact of tobacco control policies prescribed by the WHO FCTC Framework Convention on Tobacco control.

## CONFLICTS OF INTEREST

The authors have completed and submitted the ICMJE Form for Disclosure of Potential Conflicts of Interest and none was reported.
